# A Mobile Device App to Reduce Medication Errors and Time to Drug Delivery During Pediatric Cardiopulmonary Resuscitation: Study Protocol of a Multicenter Randomized Controlled Crossover Trial

**DOI:** 10.2196/resprot.7901

**Published:** 2017-08-22

**Authors:** Johan N Siebert, Frederic Ehrler, Christian Lovis, Christophe Combescure, Kevin Haddad, Alain Gervaix, Sergio Manzano

**Affiliations:** ^1^ Department of Pediatric Emergency Medicine Geneva Children’s Hospital University Hospitals of Geneva Geneva Switzerland; ^2^ Division of Medical Information Sciences Department of Radiology and Medical Informatics University Hospitals of Geneva Geneva Switzerland; ^3^ Division of Clinical Epidemiology Department of Health and Community Medicine University of Geneva and University Hospitals of Geneva Geneva Switzerland

**Keywords:** resuscitation, medication errors, pharmaceutical preparations, pediatrics, biomedical technology, children, emergency, simulation

## Abstract

**Background:**

During pediatric cardiopulmonary resuscitation (CPR), vasoactive drug preparation for continuous infusions is complex and time-consuming. The need for individual specific weight-based drug dose calculation and preparation places children at higher risk than adults for medication errors. Following an evidence-based and ergonomic driven approach, we developed a mobile device app called Pediatric Accurate Medication in Emergency Situations (PedAMINES), intended to guide caregivers step-by-step from preparation to delivery of drugs requiring continuous infusion. In a prior single center randomized controlled trial, medication errors were reduced from 70% to 0% by using PedAMINES when compared with conventional preparation methods.

**Objective:**

The purpose of this study is to determine whether the use of PedAMINES in both university and smaller hospitals reduces medication dosage errors (primary outcome), time to drug preparation (TDP), and time to drug delivery (TDD) (secondary outcomes) during pediatric CPR when compared with conventional preparation methods.

**Methods:**

This is a multicenter, prospective, randomized controlled crossover trial with 2 parallel groups comparing PedAMINES with a conventional and internationally used drug infusion rate table in the preparation of continuous drug infusion. The evaluation setting uses a simulation-based pediatric CPR cardiac arrest scenario with a high-fidelity manikin. The study involving 120 certified nurses (sample size) will take place in the resuscitation rooms of 3 tertiary pediatric emergency departments and 3 smaller hospitals. After epinephrine-induced return of spontaneous circulation, nurses will be asked to prepare a continuous infusion of dopamine using either PedAMINES (intervention group) or the infusion table (control group) and then prepare a continuous infusion of norepinephrine by crossing the procedure. The primary outcome is the medication dosage error rate. The secondary outcome is the time in seconds elapsed since the oral prescription by the physician to drug delivery by the nurse in each allocation group. TDD includes TDP. Stress level during the resuscitation scenario will be assessed for each participant by questionnaire and recorded by the heart rate monitor of a fitness watch. The study is formatted according to the Consolidated Standards of Reporting Trials Statement for Randomized Controlled Trials of Electronic and Mobile Health Applications and Online TeleHealth (CONSORT-EHEALTH) and the Reporting Guidelines for Health Care Simulation Research.

**Results:**

Enrollment and data analysis started in March 2017. We anticipate the intervention will be completed in late 2017, and study results will be submitted in early 2018 for publication expected in mid-2018. Results will be reported in line with recommendations from CONSORT-EHEALTH and the Reporting Guidelines for Health Care Simulation Research .

**Conclusions:**

This paper describes the protocol used for a clinical trial assessing the impact of a mobile device app to reduce the rate of medication errors, time to drug preparation, and time to drug delivery during pediatric resuscitation. As research in this area is scarce, results generated from this study will be of great importance and might be sufficient to change and improve the pediatric emergency care practice.

**Trial Registration:**

ClinicalTrials.gov NCT03021122; https://clinicaltrials.gov/ct2/show/NCT03021122 (Archived by WebCite at http://www.webcitation.org/6nfVJ5b4R)

## Introduction

While many drugs can be directly injected without prior preparation, others require a fastidious and complicated preparation and dilution by nurses before administration as continuous infusions. During pediatric cardiopulmonary resuscitation (CPR), quick, accurate, and safe preparation and administration of intravenous (IV) vasoactive drugs for continuous infusion is both complex and time-consuming [1-3]. In some critical situations such as postcardiac arrest return of spontaneous circulation (ROSC) or septic shock, preparing those drugs is particularly challenging. Contrarily to adults, children require individual specific weight-based drug dose calculation and preparation. The lower dosing-error tolerance [4] places children at higher risk than adults for life-threatening errors [5-7]. Medication errors have been reported in up to 41% of pediatric resuscitations, the most common being incorrect medication dosage, found in up to 65% of cases [8]. Proper preparation and delivery of these drugs could favorably affect pediatric resuscitation outcomes.

In resuscitations, time is also a decisive success criterion. It is well established that during the first 15 minutes of pediatric resuscitation, survival and favorable neurological outcome decrease linearly by 2.1% and 1.2% per minute, respectively [9]. They are negatively affected by time to drug preparation (TDP) and time to drug delivery (TDD) [10]. In a study with adults in cardiac arrest, the chance of ROSC was decreased by 4% for every 1-minute delay in delivery of a vasopressor [11].

To address these problems, we followed an evidence-based and ergonomic approach [12] to develop an innovative and customizable mobile device app called Pediatric Accurate Medication in Emergency Situations (PedAMINES). This app was designed to support nurses and physicians step-by-step from order to delivery of a wide range of drugs in real time, including those requiring continuous infusion [13]. In a previous single center simulation-based randomized controlled trial, we have shown that medication errors reduced significantly from 70% to 0% by using PedAMINES when compared with conventional methods [14]. PedAMINES also dramatically reduced TDP and TDD. The purpose of this protocol is to investigate whether the use of PedAMINES might similarly reduce medication errors, TDP, and TDD in other university hospitals and in smaller hospitals where nurses and physicians are exposed to a much lower extent to pediatric resuscitations. Simulation was used as an investigational method to assess these outcomes. We hypothesize that PedAMINES might reduce medication errors and delays to TDP and delivery independently of the existing conventional preparation methods or nurse skills. We expect even bigger improvements in smaller hospitals due to strong disparities between nurses less exposed to pediatric resuscitations.

## Methods

### Study Design

**Figure 1:**
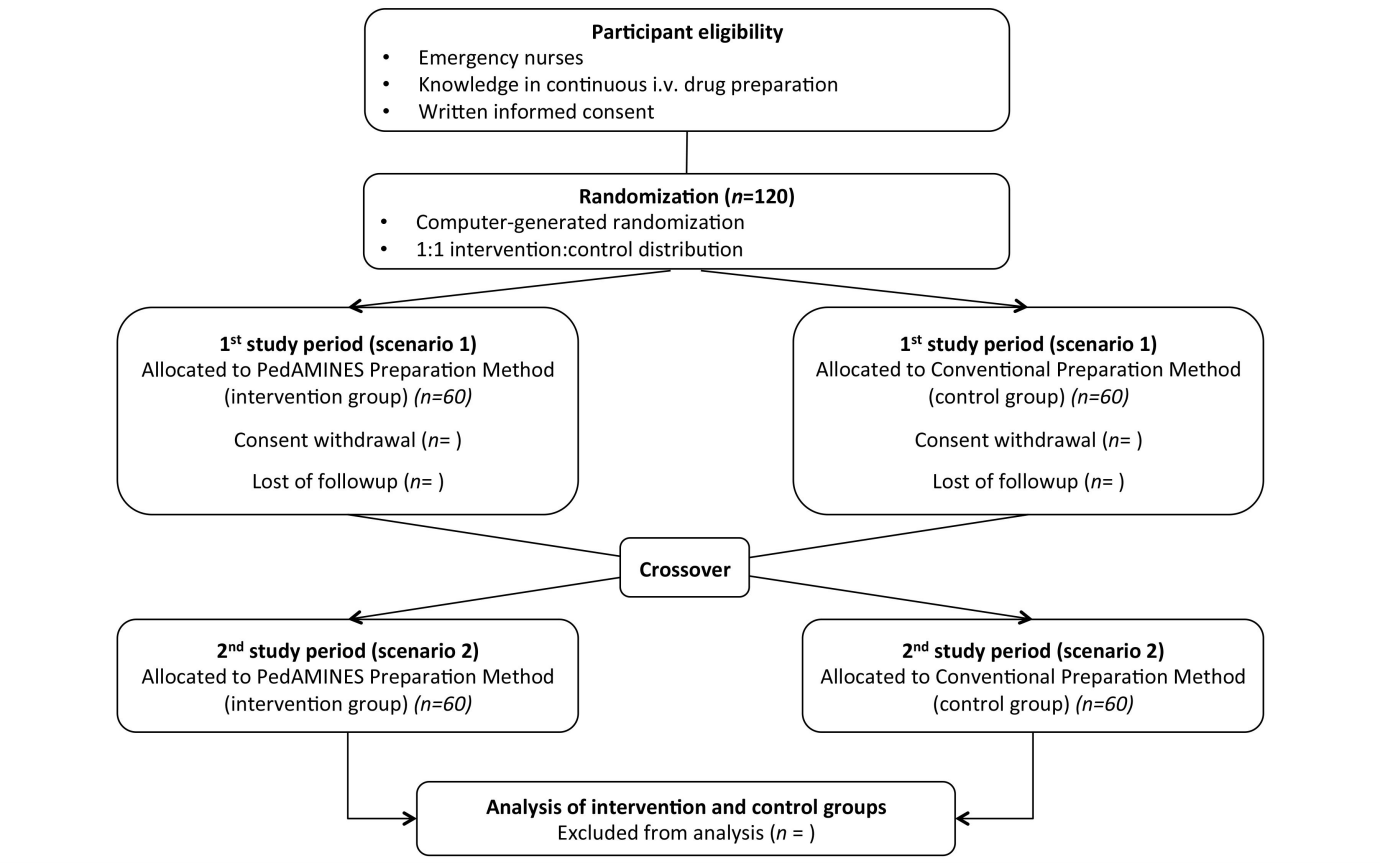
Study design and flowchart per study center.

**Figure 2:**
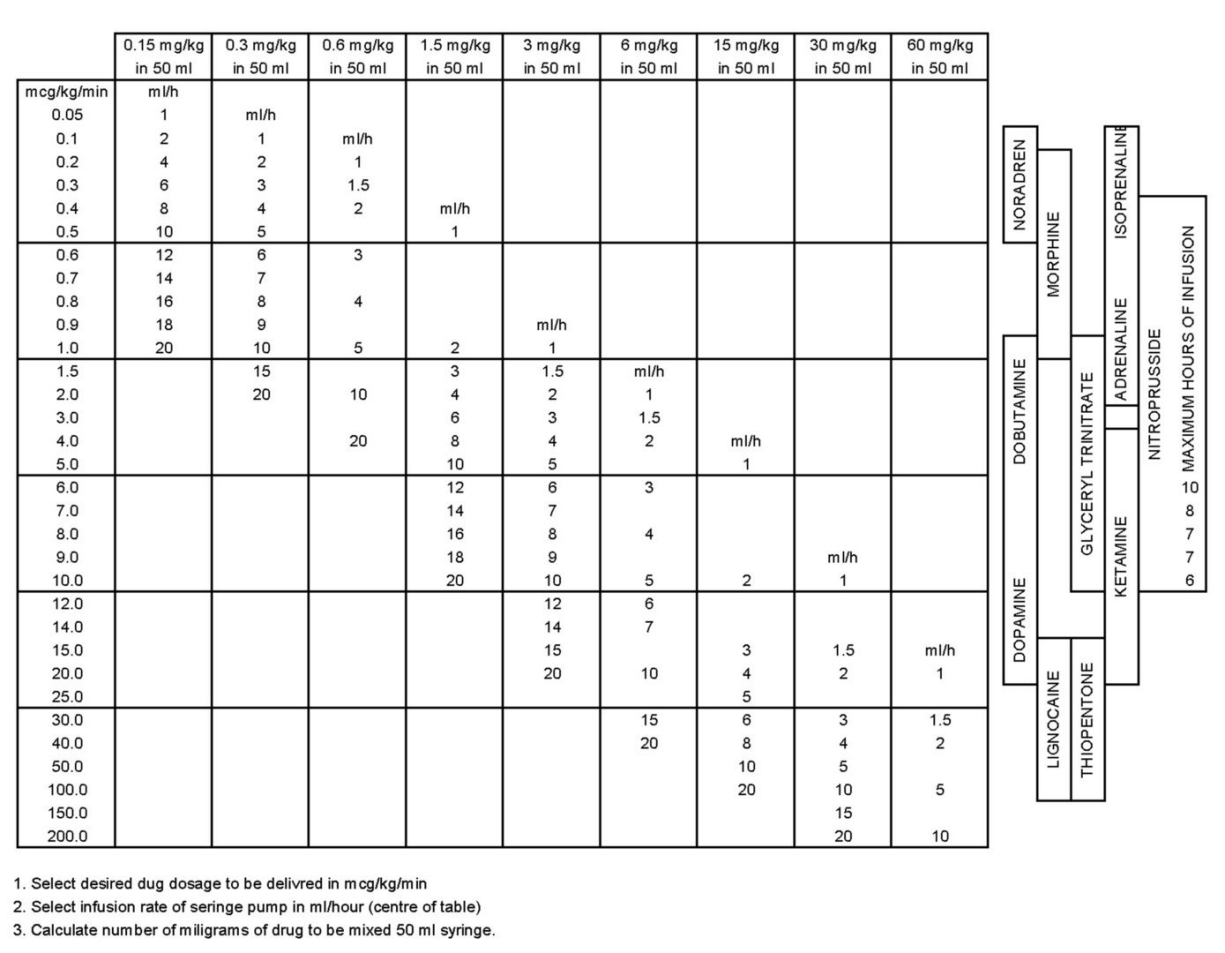
Calculation of the Composition of Drug Infusions (Syringe Pump). Frank Shann Drug Doses [15].

The study is a prospective, multicenter, randomized controlled crossover trial with 2 parallel groups (Figure 1) comparing PedAMINES [13,14] with a conventional and internationally used drug infusion rate table method [15] (derived from the rule of six [16]) in the preparation of continuous drug infusion during a standardized simulation-based pediatric postcardiac arrest scenario. The infusion rate table is presented as a spreadsheet, enabling the preparation of the commonly ordered concentrations of vasoactive drugs at varying dose ranges based on the patient’s weight (Figure 2). To calculate the composition of the drug infusion, one first selects the desired drug dosage to be delivered in μg/kg/min (first column). The next step is to select the initial infusion rate in mL/h (center of the table). Finally, one calculates the milligrams of drug based on the weight of the patient to be diluted with compatible fluids (sodium chloride 0.9%, etc) in a total volume of 50 mL (first row).

The study is formatted according to the Consolidated Standards of Reporting Trials Statement for Randomized Controlled Trials of Electronic and Mobile Health Applications and Online TeleHealth (CONSORT-EHEALTH) [17] and the Reporting Guidelines for Health Care Simulation Research [18].

### Selection of Participants

Certified nurses are eligible for inclusion in this study. Participants must know how to prepare continuous intravenous drug injections, have previously completed a standardized 5-minute introductory course to the use of PedAMINES dispensed by an investigator of the study (SM), and be willing and able to grant written informed consent. Written informed consent will be obtained from all the participants before their voluntary involvement. Participants will be excluded if they have previously used a numeric device aimed at helping vasoactive drug preparation. Shift-working nurses will be randomly recruited on the day of the study by a blinded noninvestigator.

### Setting

**Figure 3:**
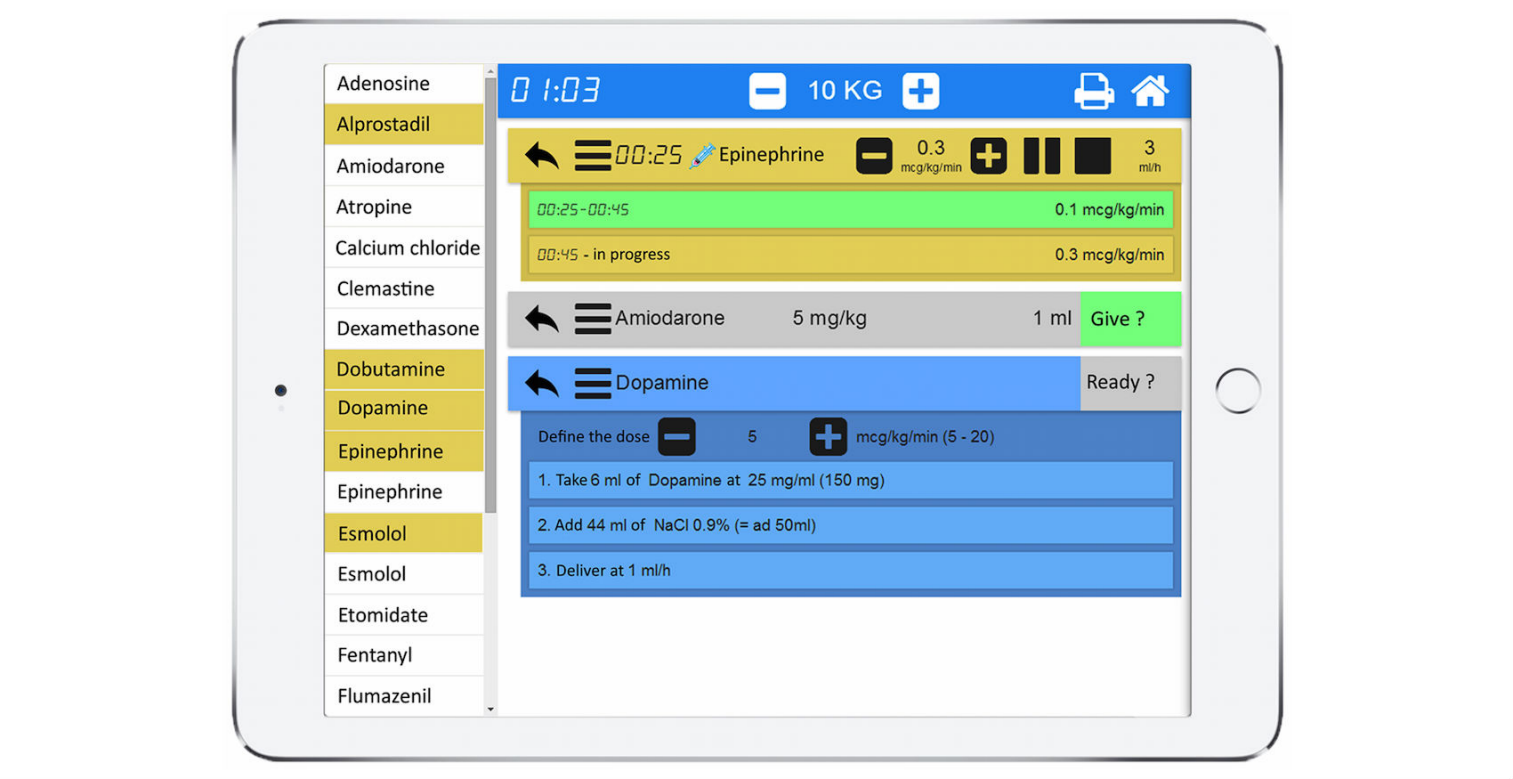
PedAMINES screenshot.

The study will be conducted in 3 university hospitals and 3 smaller hospitals with a total of approximately 150,000 visits per year. The development of PedAMINES followed a user-centered and evidence-based [12] approach with emergency department (ED) caregivers as well as software developers and ergonomists. Based on pediatric resuscitation observations and focus groups, the team worked closely together to identify the key functionalities and processes to be implemented [13]. The PedAMINES app lists all the available resuscitation drugs with doses automatically adapted to the weight or age of the patient based on information entered when starting the app. At the time of the study, 15 drugs for continuous infusion and 19 drugs for direct IV injection will be listed in the PedAMINES app and at the nurse’s disposal. By a simple touch, any of the listed drugs can be selected and preparation detailed according to a standardized and simplified path. In the case of a continuous infusion, this path is composed of 3 steps: (1) drug selection, (2) dilution of the initial drug concentration, and (3) conversion of the prescribed dose rate in μg/kg/min into infusion pump rate in mL/h. For each drug, the exact amount to prepare is clearly displayed and thus avoids the necessity for calculations (Figure 3). This is based on the app’s ability to automatically calculate the optimal weight-based final infusion pump rate and describe the preparation sequence required to achieve it independently of the nurse competency in this domain. The nurse may at any time interact with the app. The user can start, pause, stop, increase, or diminish the perfusion rate. Multiple drugs can be prepared and run in parallel. All actions performed by the nurses will be sequentially saved locally on the device in historic files to preserve information that can be retrieved at any time for debriefing or medicolegal purposes. Historic files can also be erased or safely exported and saved in institutional electronic health records.

### Intervention

The scenario in this study is a short, approximately 15-minute, standardized highly realistic pediatric resuscitation simulation on a high-fidelity manikin (Laerdal SimJunior). SimJunior represents a realistic 6-year-old boy and simulates a wide range of conditions from a healthy, talking child to an unresponsive, critical patient with no vital signs. This simulator provides all functionalities that are relevant to assess the research questions, including chest compression and ventilation and vascular access, as well as realistic and interactive vital signs. To date, high-fidelity simulation has become essential to study resuscitations skills and technologies that cannot be practiced during CPR because interindividual diversity among patients and their diseases make CPR studies hard to standardize in critical situations [19]. Moreover, by standardizing the scenario and environment, we will avoid effect modifiers by preventing the influence of undesired variables on the outcomes.

Consistent with standard emergency medicine practice, we will create resuscitation teams. Two members from the study team, remaining the same along the whole study period, will assist the study participant. A Pediatric Advanced Life Support (PALS) instructor-certified emergency pediatrician will lead the resuscitation, and a nurse will assist with resuscitation by performing chest compressions and bag-valve mask ventilation according to the pediatrician instructions. Participants will be informed before the scenario starts that these 2 people are study team members. Both study team members will guide each participant through a series of predefined key steps, blinded to the participant, following a standardized resuscitation scenario (Multimedia Appendix 1). The physician will order the medications and allow progression through the scenario only once predefined milestones have been reached, regardless of the occurrence of errors or time to achieve them. The study-specific training and standardization of both study team members is ensured through their previous involvement in the pilot study [14] and by following the predefined scenario. A certified technician will operate the simulator. The study will take place during a 1-week period in every hospital and will occupy each nurse for a single 30-minute period. Thus, we anticipate a very low rate of drop-outs or loss of follow-up. To ensure the presence of participants on the day of participation, shift-working nurses will be randomly recruited 1 month before the start of the study by a blinded noninvestigator. They will be informed of the upcoming simulation study but not of its purpose and outcomes.

On the day of participation, nurses will complete a survey collecting data regarding their demographics, nursing training, and simulation and computer experience. After random allocation, each participating nurse will receive a standardized 5-minute training session on how to use the PedAMINES app. Nurses will remain unaware of specific endpoints of the study during this learning session. The simulation manikin characteristics are then presented. The nurses will then be asked to perform the pediatric resuscitation scenario, including post-ROSC. This scenario is standardized to follow the same chronological progression and range of difficulty to ensure each participant is exposed to exactly the same case, with similar challenges in decision making and treatment preparation provided on the same manikin. The uniform delivery of the scenario all along the study will minimize confounders. Study team members will only adapt to progression speed of participants through the scenario by maintaining a stressful resuscitation atmosphere. The scenario will be videorecorded and conducted in situ in the pediatric resuscitation rooms of each collaborating center to increase realism. High levels of realism are known to immerse participants in the simulated experience and prevent confounding variables that might potentially affect the way individuals perform [19]. On the day of participation, the resuscitation room will be exclusively devoted to the simulation to prevent unexpected interruption or external stimuli. Monitoring alarms will be activated to increase realism. All resuscitation equipment in the resuscitation room will be at the disposal of the nurse. In both allocation groups, the decision to use or not use any equipment will remain personal as in real life. Neither pilot testing nor repetitions will be permitted. There will be neither interventions nor educational adjuncts prior to or after the study period. Once the experiment is completed, the nurse will be required to recall and describe precisely how she prepared dopamine and norepinephrine in order to verify that the drug names and original doses prescribed by the physician were correctly understood to assess the presence of comprehension bias. Then, a quick oral debriefing, shorter than 5 minutes, on how the drugs were actually prepared and how they should have been prepared without errors will be provided to each participant by one of the study team members. The debriefing will focus on both preparation methods.

### Outcome Measures

The primary outcome of the study is the medication dosage error rate in each allocation group during the sequence from drug preparation to drug injection. A secondary outcome is the elapsed time in seconds, in each allocation group, between the oral prescription by the physician and drug delivery by the nurse. TDP completion by the nurse is included in TDD. Comparisons of medication errors and TDD between allocation groups in each hospital and between each of the 6 hospitals will be assessed as secondary outcomes. At the end of the scenario, a 4-item questionnaire using a 10-point Likert scale will be provided to the participants. The questionnaire measures (1) the overall stress perceived (On a scale of 1 to 10, how much stress did you feel during the whole resuscitation scenario?), (2) the stress perceived with PedAMINES (On a scale of 1 to 10, how much stress did you feel using PedAMINES?), (3) the stress perceived with the infusion rate table (On a scale of 1 to 10, how much stress did you feel using the infusion rate table?), and (4) the satisfaction about the preparation method used during the resuscitation scenario (On a scale of 1 to 10, how much satisfaction did you get during the resuscitation scenario with the help of PedAMINES and with the help of the infusion rate table?). A baseline heart rate (HR) before the beginning of the scenario as well as continuous HR monitoring of each participant reflecting his or her stress level will be recorded during the resuscitation scenario. Mean delta HR values (difference between HR peak values and baseline HR) will be obtained during some small segments of scenario and correlated to the scenario phases and the preparation methods used. The segments of interest are (1) when prompted to start the resuscitation because of asystole just before massage and ventilation, (2) when prompted to prepare dopamine either with PedAMINES or the infusion rate table, (3) the first 30 seconds when dopamine is being prepared, (4) the last 30 seconds when dopamine is being prepared, (5) the setting up of the pump, (6) the washout period, (7) when prompted to prepare norepinephrine either with PedAMINES or the infusion rate table, (8) the first 30 seconds when norepinephrine is being prepared, (9) the last 30 seconds when norepinephrine is being prepared, (10) at announcement of successful resuscitation achievement, and (11) 1 minute later. Acceptability and usability testing of the app will be assessed using a 52-item questionnaire based on the unified theory of acceptance and use of technology (UTAUT) model [20]. UTAUT provides a useful tool to assess the likelihood of success for new technology introductions and helps to understand the drivers of its acceptance.

### Methods of Measurement and Data Collection

Final delivered drug concentration in μg/mL and infusion rate in μg/kg/min, TDP, and TDD will be collected during the scenario. All the actions (ie, primary and secondary outcomes) performed by the nurses during the scenario will be automatically recorded and stored by the responsive simulator detectors (Leardal SimJunior) and by 3 GoPro Hero 5 Black edition (GoPro Inc) video cameras. To avoid assessment bias, 2 evaluators will then independently review these video recordings. In case of disagreement, a third independent evaluator will help reach a consensus. The setup of the 3 cameras will be standardized to record at a resolution of 1080p, at 25 frames per second, wide field of view, and with a 16:9 aspect ratio. Cameras position will be standardized. The first camera will be placed in a harness on the nurse’s chest with an inclination of 10° downwards to film the front scene. The second camera will be placed on a tripod in front of the nurse, slightly above the head height, with an inclination of 90° downwards to film the bench where the drugs will be prepared. The third camera will be placed on a tripod 1 meter away from the nurse on his or her left (if right-handed) or right (if left-handed) and at the level of the navel to film the scene on the side. All actions performed with PedAMINES will be automatically saved locally in log files for further analysis. The validity and reliability of the app has been assessed in a prior study [14]. Stress level of each participant will be recorded during the entire resuscitation scenario with the HR monitor on a Polar A360 watch (Polar Electro Oy). The data will be stored on the wristwatch itself with further analysis being accomplished offline. Data collection will be carried out using Excel spreadsheet version 2011 (Microsoft Corp).

This study offers the great advantage of being very short in duration, around 30 minutes per nurse. Therefore, neither follow-up nor retention plans will be necessary. The intervention protocol is highly standardized, and nurse deviation from protocol in terms of drug preparation is a parameter that is of interest in our study (ie, in terms of medication errors or delays in drug preparation).

### Sample Size

The primary objective of this study is to detect a difference in rates of medication errors between groups (with PedAMINES vs without PedAMINES). In our crossover pilot study conducted in a single university hospital, the rate of medication errors was 70% (14/20, 95% CI 46%-88%) without PedAMINES and 0% (0/20, 95% CI 0%-17%) with PedAMINES [14]. We aim to reproduce these results in 3 university hospitals and 3 smaller hospitals. The sample size was calculated to have a power of 90% to detect a difference of at least 30% in rates of medication error between intervention groups in both sets of hospitals (university and smaller hospitals). We consider that a difference of 30% in the rate of medication errors is sufficient to modify the practice. We assumed a rate of error of 15% with PedAMINES and 45% without PedAMINES. Since this study is a crossover, observations will be paired and a McNemar test will be used to compare the rate of errors between intervention groups. For the sample size calculation, we assumed that 5% of nurses will commit a medication error with PedAMINES but not without PedAMINES and that 35% of nurses will commit a medication error without PedAMINES but not with PedAMINES (Multimedia Appendix 2). With a 2-sided risk alpha of 0.05, the needed sample size is 43 nurses for each set of hospitals. Finally, 8 nurses per randomized group must be recruited in each participating center (16 nurses per center). To prevent a potential loss of power due to misspecification of assumptions, 10 nurses will be recruited per randomized group and per participating center (total sample size: 120 nurses).

### Randomization and Blinding

Nurses will be randomized using a stratified, single, constant 1:1 allocation ratio determined with Web-based software [21]. Blinding to the purpose of the study during recruitment will be maintained to minimize preparation bias. Nurses will be unblinded after randomization. Allocation concealment will be ensured with sealed envelopes and will not be released until the nurses start the scenario. The study team members will be revealed just before the scenario starts, and videoreviewing will be done without blinding by both video reviewers but independently and blindly from one another.

### Statistical Analysis

#### Primary Outcome

The rate of medication errors is the proportion of nurses committing a medication error. The rate of medication errors will be reported with 95% CI (Clopper-Pearson method) with each method and by study period to investigate a potential carryover effect. The error rates for each method will be compared using McNemar test for paired data. Differences in error rates will be reported with 95% CIs. Potentially, the efficacy of PedAMINES can be different according to the first method used in the crossover design (PedAMINES first: group A or the conventional preparation method first: group B). To investigate this potential effect, the difference in error rates (PedAMINES vs conventional method) will also be reported by randomized group (paired observations) and by period (independent observations). McNemar, chi-square, or Fisher exact test will be used to compare interventions. Errors will also be measured as the deviation in percent from the amount of delivered drug compared with the original dose prescribed by the physician. Absolute deviation will be analyzed. The mean (SD) difference in deviation obtained with each method will be reported with 95% CI. A *t* test for paired data will be used to compare interventions. Mean differences will be also reported by randomized group and by crossover period. We will assess the model for the presence of carryover and period effects following the Hills-Armitage approach to crossover study analysis [22]. The analysis of the primary outcome will be conducted for the university hospitals and for smaller hospitals.

#### Secondary Outcomes

For TDD and TDP, the mean times will be reported with 95% CI for each arm and each study period to investigate a potential carryover effect. In case of a carryover effect, intervention arms will be compared within each study period using *t* tests for independent groups. If no carryover effect is found, data will be paired and analyzed using *t* tests for paired data.

For the primary and secondary outcomes, logistic regression analyses will be conducted to test a difference in rates of errors between university hospitals and smaller hospitals with PedAMINES and with the conventional preparation method. In a generalized estimating equation logistic regression model, an interaction between interventions and university/smaller hospitals will be tested to investigate a potential modification of the efficacy of PedAMINES in smaller hospitals compared with university hospitals. Analyses of primary and secondary outcomes will be conducted in nurses with more than 10 years of experience and in nurses with less than 10 years of experience. Means and standard deviations will be determined for stress and satisfaction scores of individuals for each questionnaire item as well as for the UTAUT questionnaire and reported with descriptive statistics. Pearson correlations will be computed between the HR measures obtained with the watch and the scenario phases for each of the preparation methods used. Data analysis will be carried out using GraphPad Prism version 6.0 (GraphPad Software, Inc) for graph figures, Stata/IC version 14 (StataCorp LLC) for descriptive analyses, and R version 2.15.2 (R Foundation) for statistical tests and 95% CI. Due to the nature of the interventions, we expect to have no missing data. In case of missing data, a complete case analysis will be conducted and no multiple imputation is planned. In order to assess interrater reliability on video reviewing, a kappa score—which provides a measure of interrater agreement independent of chance—will be calculated.

### Ethics and Informed Consent

Ethical approval has been obtained from the institutional ethics committee, and the trial was registered at ClinicalTrials.gov [NCT03021122]. The study will be conducted in accordance with the principles of the Declaration of Helsinki, the standards of Good Clinical Practice, and Swiss regulatory requirements.

## Results

Enrollment and data analysis started in March 2017. We anticipate that the intervention will be complete in late 2017, and study results will be submitted in early 2018 for publication expected in mid-2018. Results will be reported in line with recommendations in the CONSORT-EHEALTH [17] and the Reporting Guidelines for Health Care Simulation Research [18].

## Discussion

### Principal Findings

Despite many advances in the medical field in recent years and in particular in emergency medicine, suboptimal quality of resuscitation is still common for both adult and pediatric patients [23]. Currently, the median hospital survival rate from pediatric in-hospital cardiopulmonary arrest is 36% [23], whereas it is below 10% for out-of-hospital cardiopulmonary arrest [24,25]. Survival from resuscitation is time-sensitive and relies in part on administration of certain drugs without delay [10]. While many drugs can be directly injected without prior preparation, others require correct, precise, and fast preparation and dilution by nurses before administration as a continuous infusion. The latter warrants titration of the drugs and the maintenance of steady blood levels within therapeutic ranges. However, despite the availability of conversion methods intended to simplify the infusions such as infusion rate tables or nomograms [26], these methods remain difficult to use and infusions subject to medication errors.

In a recent randomized trial in a pediatric ED, the use of a reference book providing weight-based precalculated doses was associated with a lower proportion of prescribing errors for drugs administered by infusion [27]. The study, however, didn’t look at the preparation errors. Errors with infusions frequently result from mistakes during preparation due to wrong drug-volume calculations, imprecision with volume measurements, or incorrect mixing during dilution [1,28,29]. Children are particularly at risk for such medication errors because they require individual specific weight-based drug dose calculation and preparation. Moreover, disruptive anxiety and exogenous conditions encountered during resuscitation increase the nurse’s cognitive workload and may add to the risk of errors. At this stage, even small errors either in drug calculation or infusion pump flow rate may have a large detrimental impact on the amount of drug delivered [30-32]. This can be deleterious to critically ill and unstable patients [7].

Being able to reduce medication errors and TDD in resuscitation is imperative. If ROSC is quickly achieved and maintained after the onset of cardiac arrest, survival might be improved [33] since early hemodynamic optimization improves patient outcome [34,35].

Some authors have advocated replacing as much as possible tasks inducing cognitive load during pediatric resuscitation by automated actions in order to optimize patient care and diminish medication errors [36,37]. Numerous interventions involving information technologies have been developed to improve the security of the 3 major steps of medication process: prescription, compounding, and administration [38]. However, apart from computerized physician order entry systems, few robust data are available to measure their real impact on patient safety [39]. In addition, there have been few studies assessing information technologies during resuscitation in both adults and pediatric patients. In particular, there has been no multicenter randomized controlled trial evaluating the impact of a mobile app to reduce medication errors and decrease TDP and TDD during pediatric resuscitation. As research in this area is scarce, it is anticipated that the results generated from this study will therefore be of great importance and might be sufficient to change and improve the pediatric emergency care practice. Given that most of the results obtained from simulation-based resuscitation studies agree with those obtained from studies in real life, we are confident that PedAMINES could be of great interest for real situations.

### Limitations

A limitation of this study is that it will be conducted during a resuscitation simulation-based scenario. This choice was related to the ethical and organizational difficulties of conducting studies with patients in critical situations. However, several studies have demonstrated the benefit of simulation as an investigative research methodology to answer research questions that otherwise could not be answered during resuscitation [19]. Simulation-based CPR scenarios may overcome these limitations by providing a standardized and controlled environment, detailed feedback analysis of the resuscitation stages using audiovisual recordings, and reproducibility. To date, high-fidelity simulation has become essential to study resuscitations skills or technologies.

Our study is not intended to compare PedAMINES with smart IV pumps. As recently reported, there is no conclusive evidence showing that smart pumps prevent medication errors and adverse drug events [40,41]. In addition, little is known about the kind of errors that still occur with their use. Moreover, the lack of specialized pharmacy facilities in many smaller hospitals or in other countries around the world limit their use. PedAMINES does not have these limitations for use and can be used in smaller hospitals and worldwide.

It should be noted that the Likert-type questionnaire used in this study to measure stress has not been assessed for validity, internal consistency, reliability, or generalizability. Although it cannot objectively measure the stress perceived, it can be used to measure the difference of perceived stress.

## Appendix

Multimedia Appendix 1 Detailed description of the resuscitation scenario used during the simulation.

Multimedia Appendix 2 Contingency table for the expected McNemar test comparing the rate of errors between intervention groups. Percentages in bold denote the values hypothesized for the sample size calculation.

Multimedia Appendix 3 Peer-review document: Acceptance letter by the Swiss National Science Foundation.

## Abbreviations

CONSORT-EHEALTH: Randomized Controlled Trials of Electronic and Mobile Health Applications and Online TeleHealth
CPR: cardiopulmonary resuscitation
ED: emergency department
HR: heart rate
IV: intravenous
PALS: Pediatric Advanced Life Support
PedAMINES: Pediatric Accurate Medication in Emergency Situations
ROSC: return of spontaneous circulation
TDD: time to drug delivery
TDP: time to drug preparation
UTAUT: unified theory of acceptance and use of technology

## References

[1] Larsen GY, Parker HB, Cash J, O'Connell M, Grant MC (2005). Standard drug concentrations and smart-pump technology reduce continuous-medication-infusion errors in pediatric patients. Pediatrics.

[2] Moyen E, Camiré E, Stelfox HT (2008). Clinical review: medication errors in critical care. Crit Care.

[3] Polischuk E, Vetterly CG, Crowley KL, Thompson A, Goff J, Nguyen-Ha P, Modery C (2012). Implementation of a standardized process for ordering and dispensing of high-alert emergency medication infusions. J Pediatr Pharmacol Ther.

[4] Kaushal R, Bates DW, Landrigan C, McKenna KJ, Clapp MD, Federico F, Goldmann DA (2001). Medication errors and adverse drug events in pediatric inpatients. JAMA.

[5] Gonzales K (2010). Medication administration errors and the pediatric population: a systematic search of the literature. J Pediatr Nurs.

[6] Hoyle JD, Davis AT, Putman KK, Trytko JA, Fales WD (2012). Medication dosing errors in pediatric patients treated by emergency medical services. Prehosp Emerg Care.

[7] Kaufmann J, Laschat M, Wappler F (2012). Medication errors in pediatric emergencies: a systematic analysis. Dtsch Arztebl Int.

[8] Porter E, Barcega B, Kim TY (2014). Analysis of medication errors in simulated pediatric resuscitation by residents. West J Emerg Med.

[9] Matos RI, Watson RS, Nadkarni VM, Huang H, Berg RA, Meaney PA, Carroll CL, Berens RJ, Praestgaard A, Weissfeld L, Spinella PC (2013). Duration of cardiopulmonary resuscitation and illness category impact survival and neurologic outcomes for in-hospital pediatric cardiac arrests. Circulation.

[10] Andersen LW, Berg KM, Saindon BZ, Massaro JM, Raymond TT, Berg RA, Nadkarni VM, Donnino MW (2015). Time to epinephrine and survival after pediatric in-hospital cardiac arrest. JAMA.

[11] Hubble MW, Johnson C, Blackwelder J, Collopy K, Houston S, Martin M, Wilkes D, Wiser J (2015). Probability of return of spontaneous circulation as a function of timing of vasopressor administration in out-of-hospital cardiac arrest. Prehosp Emerg Care.

[12] Ehrler F, Haller G, Sarrey E, Walesa M, Wipfli R, Lovis C (2015). Assessing the usability of six data entry mobile interfaces for caregivers: a randomized trial. JMIR Hum Factors.

[13] Hagberg H, Siebert J, Gervaix A, Daehne P, Lovis C, Manzano S, Ehrler F (2016). Improving drugs administration safety in pediatric resuscitation using mobile technology. Stud Health Technol Inform.

[14] Siebert JN, Ehrler F, Combescure C, Lacroix L, Haddad K, Sanchez O, Gervaix A, Lovis C, Manzano S (2017). A mobile device app to reduce time to drug delivery and medication errors during simulated pediatric cardiopulmonary resuscitation: a randomized controlled trial. J Med Internet Res.

[15] Shann F (2014). Drug Doses RCH Intensive Care Unit, 16th Edition.

[16] McLeroy PA (1994). The rule of six: calculating intravenous infusions in a pediatric crisis situation. Hosp Pharm.

[17] Eysenbach G (2011). CONSORT-EHEALTH: improving and standardizing evaluation reports of Web-based and mobile health interventions. J Med Internet Res.

[18] Cheng A, Kessler D, Mackinnon R, Chang TP, Nadkarni VM, Hunt EA, Duval-Arnould J, Lin Y, Cook DA, Pusic M, Hui J, Moher D, Egger M, Auerbach M, International Network for Simulation-based Pediatric Innovation‚ Research‚Education (INSPIRE) Reporting Guidelines Investigators (2016). Reporting guidelines for health care simulation research: extensions to the CONSORT and STROBE statements. Simul Healthc.

[19] Cheng A, Auerbach M, Hunt EA, Chang TP, Pusic M, Nadkarni V, Kessler D (2014). Designing and conducting simulation-based research. Pediatrics.

[20] Venkatesh V, Morris MG, Davis GB, Davis FD (2003). User acceptance of information technology: toward a unified view. MIS Q.

[21] sealedenvelope.com.

[22] Hills M, Armitage P (1979). The two-period cross-over clinical trial. Br J Clin Pharmacol.

[23] Meaney PA, Bobrow BJ, Mancini ME, Christenson J, de Caen AR, Bhanji F, Abella BS, Kleinman ME, Edelson DP, Berg RA, Aufderheide TP, Menon V, Leary M (2013). Cardiopulmonary resuscitation quality: [corrected] improving cardiac resuscitation outcomes both inside and outside the hospital: a consensus statement from the American Heart Association. Circulation.

[24] Jayaram N, McNally B, Tang F, Chan PS (2015). Survival after out-of-hospital cardiac arrest in children. J Am Heart Assoc.

[25] Kämäräinen A (2010). Out-of-hospital cardiac arrests in children. J Emerg Trauma Shock.

[26] Sherry E, Burton GW, Wilkins DG (1993). Infusion nomograms. Anaesthesia.

[27] Larose G, Levy A, Bailey B, Cummins-McManus B, Lebel D, Gravel J (2017). Decreasing prescribing errors during pediatric emergencies: a randomized simulation trial. Pediatrics.

[28] Adapa RM, Mani V, Murray LJ, Degnan BA, Ercole A, Cadman B, Williams CE, Gupta AK, Wheeler DW (2012). Errors during the preparation of drug infusions: a randomized controlled trial. Br J Anaesth.

[29] Ameer A, Dhillon S, Peters M, Ghaleb M (2015). Systematic literature review of hospital medication administration errors in children. IPRP.

[30] Allen EM, Van Boerum DH, Olsen AF, Dean JM (1995). Difference between the measured and ordered dose of catecholamine infusions. Ann Pharmacother.

[31] Lehmann CU, Kim GR, Gujral R, Veltri MA, Clark JS, Miller MR (2006). Decreasing errors in pediatric continuous intravenous infusions. Pediatr Crit Care Med.

[32] Parshuram CS, To T, Seto W, Trope A, Koren G, Laupacis A (2008). Systematic evaluation of errors occurring during the preparation of intravenous medication. CMAJ.

[33] Neumar RW, Nolan JP, Adrie C, Aibiki M, Berg RA, Böttiger BW, Callaway C, Clark RSB, Geocadin RG, Jauch EC, Kern KB, Laurent I, Longstreth WT, Merchant RM, Morley P, Morrison LJ, Nadkarni V, Peberdy MA, Rivers EP, Rodriguez-Nunez A, Sellke FW, Spaulding C, Sunde K (2008). Post-cardiac arrest syndrome: epidemiology, pathophysiology, treatment, and prognostication. A consensus statement from the International Liaison Committee on Resuscitation (American Heart Association, Australian and New Zealand Council on Resuscitation, European Resuscitation Council, Heart and Stroke Foundation of Canada, InterAmerican Heart Foundation, Resuscitation Council of Asia, and the Resuscitation Council of Southern Africa); the American Heart Association Emergency Cardiovascular Care Committee; the Council on Cardiovascular Surgery and Anesthesia; the Council on Cardiopulmonary, Perioperative, and Critical Care; the Council on Clinical Cardiology; and the Stroke Council. Circulation.

[34] López-Herce J, del Castillo J, Matamoros M, Canadas S, Rodriguez-Calvo A, Cecchetti C, Rodríguez-Núnez A (2014). Post return of spontaneous circulation factors associated with mortality in pediatric in-hospital cardiac arrest: a prospective multicenter multinational observational study. Crit Care.

[35] Topjian AA, French B, Sutton RM, Conlon T, Nadkarni VM, Moler FW, Dean JM, Berg RA (2014). Early postresuscitation hypotension is associated with increased mortality following pediatric cardiac arrest. Crit Care Med.

[36] Luten R, Wears RL, Broselow J, Croskerry P, Joseph MM, Frush K (2002). Managing the unique size-related issues of pediatric resuscitation: reducing cognitive load with resuscitation aids. Acad Emerg Med.

[37] Moreira ME, Hernandez C, Stevens AD, Jones S, Sande M, Blumen JR, Hopkins E, Bakes K, Haukoos JS (2015). Color-coded prefilled medication syringes decrease time to delivery and dosing error in simulated emergency department pediatric resuscitations. Ann Emerg Med.

[38] Bonnabry P (2005). Information technologies for the prevention of medication errors. CHIMIA Int J Chem.

[39] Acheampong F, Anto BP, Koffuor GA (2014). Medication safety strategies in hospitals—a systematic review. Int J Risk Saf Med.

[40] Franklin BD (2016). 'Smart' intravenous pumps: how smart are they?. BMJ Qual Saf.

[41] Schnock KO, Dykes PC, Albert J, Ariosto D, Call R, Cameron C, Carroll DL, Drucker AG, Fang L, Garcia-Palm CA, Husch MM, Maddox RR, McDonald N, McGuire J, Rafie S, Robertson E, Saine D, Sawyer MD, Smith LP, Stinger KD, Vanderveen TW, Wade E, Yoon CS, Lipsitz S, Bates DW (2016). The frequency of intravenous medication administration errors related to smart infusion pumps: a multihospital observational study. BMJ Qual Saf.

