# Supplementary material for: A Mobile Device App to Reduce Medication Errors and Time to Drug Delivery During Pediatric Cardiopulmonary Resuscitation: Study Protocol of a Multicenter Randomized Controlled Crossover Trial
Source: JMIR Res Protoc. 2017 Aug 22;6(8):e167. doi: 10.2196/resprot.7901 (PMC5585594; doi:10.2196/resprot.7901)
Supplement: Multimedia Appendix 1 [file resprot_v6i8e167_app1.pdf]

## The PedAMINES simulation-based scenario

The untimed portion of the simulation starts by turning on the 3 GoPro video cameras and the Polar A360 watch, with the nurse and the physician waiting outside the resuscitation room. Both will be invited to enter the room by the confederate nurse. When entering the resuscitation room, a clinical statement to recognize the life-threatening condition of the patient, including his weight and age, is given by the confederate nurse, as follows: "Here is Junior, a 16-kg, 3 years old boy who drowned 8 minutes ago in a pool and brought to the emergencies by his parents. He is unconscious, pale and does not breathe". At this moment, the physician asks the participating nurse to take a central pulse. Because of the invariable absence of a pulse, the nurse is asked to assist the physician to perform a 2-min full course massage and ventilation (15:2 ratio) maneuver to increase her own stress level (massage carried out by the nurse). During this time, the physician asks the confederate nurse to place a 4-derivation electrocardiogram on the manikin, an upper arm blood pressure monitor, and a digital pulse oximeter. Monitoring alarms are activated to increase realism. Then the physician asks the confederate nurse to get a peripheral vascular access on the manikin's right hand. At this time, an asystole rhythm is recognized and verbalized by the physician. On the basis of the American Heart Association (AHA) pediatric cardiac arrest algorithm for asystole, a bolus of 0.01 mg/kg epinephrine (0.1 mL/kg of 0.1mg/ml concentration) is ordered by the physician to the confederate nurse and administered by the latter. ROSC ensues with hypotension. The physician says: "He has now a return of spontaneous circulation with a pulse but with a low blood pressure. It's an hypotensive shock! This patient needs a vasoactive drug, right now!". At the end of the instruction, the nurse is then asked to prepare a 5 µg/kg/min continuous infusion of dopamine for a 16-kg boy either with the help of PedAMINES first (intervention group) or following the conventional method first (ie Shann infusion rate table [15], control group; Figure 1) and the timed scenario begins. During this time, both the physician and the confederate nurse have to maintain a stressful resuscitation atmosphere. The confederate nurse is asked to administer a 20 ml/kg sodium chloride 0.9% iv bolus. He has to evaluate/repeat the primary assessment (ABCDE approach) according to the PALS recommendations. Vital signs are frequently reported aloud.

Once the drug ready to be injected, the nurse is asked to deliver it to the patient using a syringe pump already in place. The nurse is then asked to perform a washout distraction maneuver by aspirating secretions in the throat of the manikin: "Oh no, the patient as

secretions at the back of his throat, please remove them quickly by using the yankauer!". At this moment, the crossover occurs. The physician says: "Ok, airways are now clear. But despite the volume expansion with sodium chloride and dopamine infusion, the patient is still in hypotensive shock! He needs a second vasoactive drug, right now!". The nurse is then asked to prepare a 0.1 µg/kg/min continuous infusion of norepinephrine by crossing the procedure (ie intervention group having used PedAMINES before is asked to use the conventional method, whereas control group having used the conventional method before is asked to use PedAMINES; Figure 1). At the end of the order, the nurse is asked to prepare the drug. To render the task uniform between both groups, the final volume of norepinephrine will require a decimal-point dependent calculation with both the conventional method and PedAMINES preparation methods. During this time, both the physician and the confederate nurse still have to maintain a stressful resuscitation atmosphere as described above. When the drug is ready to be injected, the nurse is asked to deliver it to the patient using a second syringe pump already in place. The beginning of the injection corresponds to the end of the scenario. The GoPro cameras and the Polar A360 watch are turned off 1 minute later.

The delivery of both drugs will require programming the same pump in a similar manner among all participants. Time elapsed after drug preparation until its delivery, that is, time needed to set up the pump, will be assessed for all participants to ensure uniformity among participants. The measured deviation between the amount of drug delivered and the actual prescribed dose will be measured by the amount of drug in the syringe. As everything is video-recorded, we will be able to verify the exact amount of drug in the syringe after the scenario.
