# Supplementary figures and images for: A Mobile Device App to Reduce Medication Errors and Time to Drug Delivery During Pediatric Cardiopulmonary Resuscitation: Study Protocol of a Multicenter Randomized Controlled Crossover Trial
Source: JMIR Res Protoc. 2017 Aug 22;6(8):e167. doi: 10.2196/resprot.7901 (PMC5585594; doi:10.2196/resprot.7901)

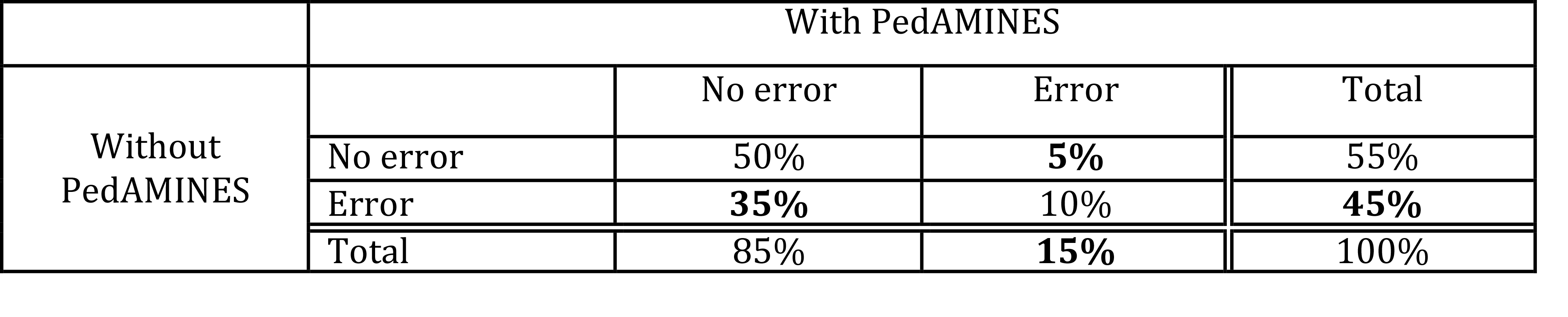

Supplement: Multimedia Appendix 2 [file resprot_v6i8e167_app2.jpg]
