# Supplementary material for: A Mobile Device App to Reduce Medication Errors and Time to Drug Delivery During Pediatric Cardiopulmonary Resuscitation: Study Protocol of a Multicenter Randomized Controlled Crossover Trial
Source: JMIR Res Protoc. 2017 Aug 22;6(8):e167. doi: 10.2196/resprot.7901 (PMC5585594; doi:10.2196/resprot.7901)
Supplement: Multimedia Appendix 3 [file resprot_v6i8e167_app3.pdf]

FNS SNF
FONDS NATIONAL SUISSE
DE LA RECHERCHE SCIENTIFIQUE

www.snf.ch
Wildhainweg 3, case postale 8232, CH-3001 Berne

Division Biologie et médecine
Tél. +41 31 308 22 22
Fax +41 31 308 21 50
E-mail div3@snf.ch

Professeur Alain Gervaix
Hôpital des Enfants
HUG
Rue Willy-Donzé 6
CH-1211 Genève 14

Berne, le 21 septembre 2016

Requête n° 32003B_169348 / 1 : décision

Monsieur,

Nous avons le plaisir de vous informer que le Conseil de la recherche vous alloue un subside de CHF 300'213.00 pour le projet de recherche « Reducing Medication Errors and Time in Vasoactive Drug Preparation and Delivery during Pediatric Resuscitation: a Multicenter, Prospective, Randomized Controlled Trial in Switzerland. ». Vous trouverez des informations complémentaires sur l'évaluation et le classement relatif de votre requête sur *my*SNF. La répartition et les conditions de l'octroi ci-jointes font partie intégrante de la décision.

Veuillez observer les dispositions du *Règlement des subsides* et du *Règlement d'exécution général relatif au règlement des subsides*. Ces documents sont à votre disposition sur le site web du FNS (cf. « Documents juridiques » ci-après). Vous trouvez également des informations détaillées sur le suivi des subsides dans les *Directives pour la gestion des projets de recherche* : www.fns.ch > le FNS > Encouragement > Documents & téléchargements > Bases juridiques. Si votre requête a été déposée en commun avec d'autres personnes, nous vous prions d'observer l'obligation d'informer les autres requérant-e-s comme mentionné aux articles 12 et 32 du *Règlement des subsides*.

En vous souhaitant plein succès dans la réalisation de votre projet, nous vous prions d'agréer, Monsieur, nos salutations distinguées.

Stéphanie Buvelot Frei

Gervaix Alain
32003B_169348 / 1
Annexe à la décision du 21 septembre 2016

Répartition du subside par rubrique

	Total	1ère tranche	2ème tranche
**Projet**			
Appareils	25'610	25'310	300
Frais de recherche	108'553	81'843	26'710
Salaires	135'000	90'000	45'000
Charges sociales	31'050	20'700	10'350
**Total**	300'213	217'853	82'360

Début: 1er janvier 2017 Durée: 18 mois

Conditions pour l'ouverture du subside:

Appareils : Accordés

Fonctions/Salaires: Accordés:

Autre(s) collaborateur(s) N. N., Assistant, 100%, 18 mois
CHF 90'000.- / 45'000.-

Charges sociales: 23% = CHF 31'050.-

Selon le "Règlement d'exécution général au règlement des subsides", l'engagement de nouveaux collaborateurs, les mutations de personnel ainsi que les ajustements de salaire nécessitent l'approbation du FNS. Ils doivent lui être soumis à l'avance avec le formulaire "Avis de mutation" via mySNF.

Subside égalité

Les jeunes chercheuses (post)doctorantes, ainsi que les chercheuses dans les hautes écoles spécialisées ne visant pas le doctorat, qui collaborent à un taux d'occupation d'au moins 60% dans des projets financés par le FNS ont droit à un subside égalité dans le cadre de mesures visant l'encouragement de carrières. Les collaboratrices doivent être employées par une institution suisse. Les ayants-droit reçoivent au maximum CHF 1'000.- par année pour de telles mesures (c'est-à-dire par tranche de 12 mois de la durée acceptée, les tranches inférieures n'étant pas imputables). Sont considérées comme mesures de développement de la carrière, le mentorat, le coaching, les cours et ateliers d'encouragement de la carrière, les rencontres et manifestations visant à tisser un réseau, etc. Le subside égalité n'est pas destiné à payer les frais de garde des enfants.

Si les frais du subside égalité ne peuvent pas être couverts par les ressources disponibles du projet, le FNS peut déclencher un paiement de compensation sur la base d'une remarque insérée dans le cadre du rapport financier final muni des pièces justificatives correspondantes.

Documents juridiques
(www.fns.ch >Encouragement > Documents & téléchargements > Bases juridiques)

- Règlement des subsides
- Règlement d'exécution général relatif au règlement des subsides

Indication des voies de droit

Conformément à l'article 13 de la loi du 14 décembre 2012 sur l'encouragement de la recherche et de l'innovation (RS 420.1), la présente décision peut faire l'objet d'un recours, dans un délai de 30 jours après sa notification, auprès du Tribunal administratif fédéral, case postale, 9023 St-Gall.

Le mémoire de recours indique les conclusions, motifs et moyens de preuve et porte la signature du ou de la recourant-e ou de son mandataire.

La décision attaquée et les pièces invoquées comme moyens de preuve, lorsqu'elles se trouvent en la possession du ou de la recourant-e, doivent être annexées à l'envoi.
